# Association Between Metabolic Syndrome Components and Vascular Structure and Function in Subjects with a Diagnosis of Long COVID: The BioICOPER Study

**DOI:** 10.3390/jcm15062348

**Published:** 2026-03-19

**Authors:** Nuria Suárez-Moreno, Leticia Gómez-Sánchez, Silvia Arroyo-Romero, Alicia Navarro-Cáceres, Andrea Domínguez-Martín, Cristina Lugones-Sánchez, Susana González-Sánchez, Andrea Sánchez-Moreno, Emiliano Rodríguez-Sánchez, Luis García-Ortiz, Manuel A. Gómez-Marcos, Marta Gómez-Sánchez, Elena Navarro-Matias

**Affiliations:** 1 Primary Care Research Unit of Salamanca (APISAL), Institute of Biomedical Research of Salamanca (IBSAL), Salamanca Primary Care Management (SACyL), Avenida de Portugal 83, 37005 Salamanca, Spain; nuria.suarez@usal.es (N.S.-M.); silvia_ar@usal.es (S.A.-R.); alicia.nav@usal.es (A.N.-C.); andreadm@usal.es (A.D.-M.); gongar04@gmail.com (S.G.-S.); andreasan@usal.es (A.S.-M.); emiliano@usal.es (E.R.-S.); 2 Castilla and León Health Service-SACYL, Regional Health Management, 37005 Salamanca, Spain; lgarciao@usal.es (L.G.-O.); enavarro@saludcastillayleon.es (E.N.-M.); 3 Emergency Service, University Hospital of La Paz P. of Castellana, 261, 28046 Madrid, Spain; leticiagmzsnchz@gmail.com; 4 Research Network on Chronicity, Primary Care and Health Promotion (RICAPPS), Avenida de Portugal 83, 37005 Salamanca, Spain; 5 Department of Medicine, University of Salamanca, Calle Alfonso X el Sabio s/n, 37007 Salamanca, Spain; 6 Department of Biomedical and Diagnostic Sciences, University of Salamanca, Calle Alfonso X el Sabio s/n, 37007 Salamanca, Spain; 7 Home Hospitalization Service, Marqués of Valdecilla University Hospital, 39008 Santander, Spain; martagmzsnchz@gmail.com

**Keywords:** long COVID, metabolic syndrome, vascular function, vascular structure, vascular ageing

## Abstract

**Background:** Long COVID is characterised by persistent symptoms after SARS-CoV-2 infection, and its impact on cardiovascular health is a growing concern. This study aimed to evaluate the association between the presence and severity of metabolic syndrome and vascular structural and functional in patients with long COVID. **Methods:** We conducted a cross-sectional study of 304 adults diagnosed with long COVID. Vascular health was assessed using carotid intima–media thickness to evaluate arterial structure, and pulse wave velocity to assess arterial stiffness. Metabolic syndrome was defined according to international criteria. Multiple regression models were performed to analyse the association between the number of metabolic syndrome components and vascular parameters, adjusting for age, sex, lifestyle and pharmacological treatments. **Results:** All vascular measures show a positive association with artery pressure. All measures except cardio–ankle vascular index were positively associated with the number of metabolic syndrome components. Carotid intima–media thickness, carotid–femoral pulse wave velocity and vascular ageing index were positively associated with waist circumference. Brachial–ankle pulse wave was positively associated with all metabolic syndrome components and showed an inverse association with HDL-cholesterol. Cardio–ankle vascular index was inversely associated with waist circumference. **Conclusions:** In conclusion, among adults with long COVID, metabolic syndrome and the accumulation of its components are associated with poorer vascular structure, function, and vascular ageing.

## 1. Introduction

Long COVID has become a relevant clinical problem due to the persistence of symptoms and/or the appearance of new manifestations beyond three months after acute SARS-CoV-2 infection, according to the WHO definition [1]. Long COVID is characterised by a complex multisystemic involvement, where interactions between the respiratory, cardiovascular and nervous systems play a fundamental role in the persistence of symptoms. For this reason, long COVID is a complex syndrome with a wide variety of prolonged and heterogeneous symptoms [2]. Furthermore, the clinical characterisation of this new entity has evolved significantly, leading to its formal classification through clinical coding systems like ICD-10, which allows for a more standardised approach to its study and diagnosis [3]. It has become a global health problem, due to its high prevalence (400 million) and annual global economic impact [4]. Beyond its functional impact, its cardiometabolic and vascular involvement is particularly concerning due to its potential medium-term prognostic implications [5]. A plausible hypothesis is that long COVID represents a condition with multiple subtypes, each with its own risk factors, biological mechanisms and disease trajectory [4]. Across these mechanisms, post-COVID endotheliopathy—predominantly microvascular with low-grade inflammation—may underlie many long COVID symptoms [6]. This may favour increased arterial stiffness and vascular ageing in individuals with long COVID, as shown in previous studies [7,8,9]. Vascular effects may also vary by sex; females with long COVID have been reported to have worse arterial elasticity [10].

In parallel, metabolic syndrome (MetS)—characterised by the co-occurrence of central obesity, elevated blood pressure, atherogenic dyslipidaemia, and impaired glucose regulation—represents a primary driver of cardiovascular risk [11,12]. MetS and its components are associated with greater arterial stiffness and subclinical structural changes; arterial stiffness is a robust measure of vascular damage and is used to estimate vascular ageing [13,14,15,16,17,18].

Pulse wave velocity (PWV) is considered a gold standard for assessing arterial stiffness, representing a lifetime integration of cumulative cardiovascular risk and serving as a robust predictor of all-cause and cardiovascular mortality [19]. However, although some studies have related MetS and its components with arterial stiffness in individuals with long COVID [20,21,22,23], future investigations are warranted to better understand the interplay between metabolic syndrome and its constituent factors and different measures of arterial stiffness and vascular ageing in long COVID, and whether these associations differ by sex. Moreover, different stiffness indices (carotid–femoral pulse wave velocity (cfPWV), brachial–ankle pulse wave velocity (baPWV) and cardio–ankle vascular index (CAVI)) may capture partially distinct dimensions of vascular impairment, justifying within-study comparisons and component-level analyses in this long COVID context. However, despite the growing evidence linking long COVID with vascular dysfunction, the extent to which pre-existing or concurrent MetS exacerbates this damage remains a critical knowledge gap. While post-COVID endotheliopathy promotes a pro-inflammatory state, MetS acts as a chronic metabolic stressor that independently drives early vascular ageing [13,18] and increased arterial stiffness [24]. The convergence of these two conditions may create a ‘synergistic’ effect on the arterial wall, significantly increasing the cardiovascular risk profile of these patients. Understanding whether the cumulative burden of MetS components translates into measurable structural and functional vascular impairment is essential for risk stratification in the long-term management of long COVID.

Therefore, within the BioICOPER study framework [24], this work aims to address these gaps by analysing the relationship of MetS—both as a clinical entity and through its individual components—with markers of vascular structure (carotid intima–media thickness), vascular function (cfPWV, baPWV, and CAVI), and overall vascular ageing. Furthermore, we sought to determine if these associations persist after adjusting for potential confounders such as pharmacological treatments and lifestyle factors. This comprehensive approach is intended to clarify the role of metabolic health as a key determinant of vascular integrity in the long COVID population.

## 2. Materials and Methods

### 2.1. Study Design

This descriptive, cross-sectional research was conducted according to [24]. The trial protocol was registered with ClinicalTrials.gov in April 2023 (identifier NCT05819840).

### 2.2. Study Cohort

**Figure 1:**
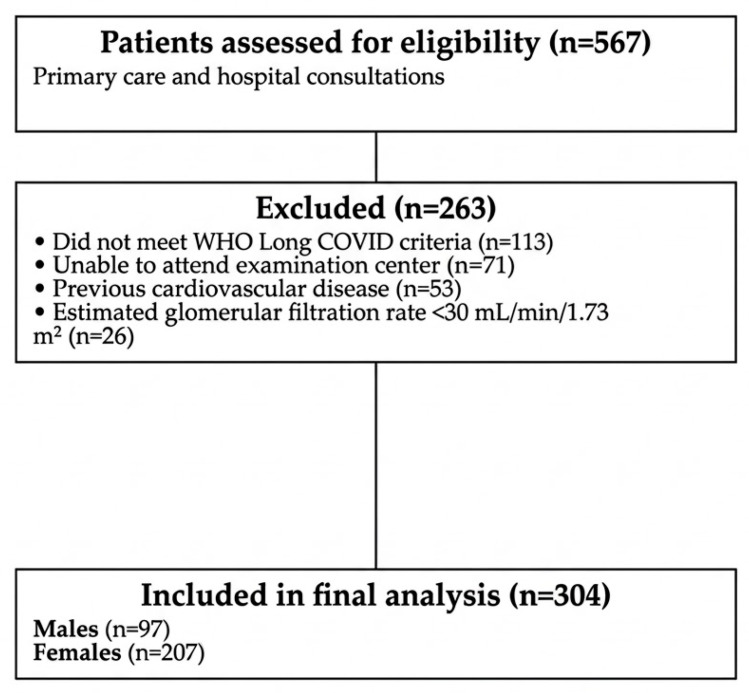
Study flowchart showing participant selection, measurements and analysis groups.

A total of 304 patients with long COVID were recruited through consecutive sampling from Primary Care clinical records and Internal Medicine outpatient clinics at the University Hospital of Salamanca, between 9 March 2023 and 10 September 2024. Due to the haste that existed in starting the recruitment of subjects and the time it took for registration, the first patients were included in the study before being approved for registration in the Clinical Trials. In accordance with WHO guidelines [1], long COVID was identified in patients with a past probable or confirmed SARS-CoV-2 infection, presenting symptoms that persist for a minimum of two months—typically evaluated three months after the initial infection—that cannot be attributed to another medical condition. These clinical manifestations might either develop after an apparent recovery or endure from the acute phase, and they can exhibit a relapsing or fluctuating pattern over time. Exclusion criteria were inability to attend the health centre due to health status, prior cardiovascular disease (e.g., ischemic heart disease or cerebrovascular events), or estimated glomerular filtration rate < 30 mL/min/1.73 m^2^. Figure 1 shows the participant flowchart for the recruitment of the participants.

### 2.3. Variables and Measurement Instruments

Data collection, encompassing both medical evaluations and questionnaires, was executed by four clinical staff members following a strictly standardised training protocol. Data quality was ensured through rigorous monitoring by an external investigator.

#### 2.3.1. Sociodemographic Variables, Lifestyle and Medical History

Age, sex, personal history of hypertension, dyslipidaemia and type 2 diabetes, and medication use were recorded. Time since acute infection was calculated as the interval between the acute infection date and study inclusion.

##### Lifestyle

Alcohol consumption was determined with a structured questionnaire, recording the amount and type of alcohol ingested during the previous 7 days measured in grams/week. We also used a standard questionnaire used in the WHO MONICA project [25] to record tobacco use.

The assessment of the Adherence to the Mediterranean Diet was carried out with the 14-question Mediterranean Diet Adherence Screener (MEDAS) questionnaire, developed by the PREDIMED group and previously validated in the Spanish population [26].

Physical activity was objectively assessed with a validated digital pedometer (Omron Hj-321 lay-UPS, Omron Healthcare Europe) [27] which measures total steps, aerobic steps, distance travelled in Km, and calories burned in the last 7 days.

#### 2.3.2. Vascular Structure and Function

##### Vascular Structure

Vascular structure was assessed by measuring the mean carotid intima–media thickness (cIMT) of the common carotid artery using a Sonosite Micromax ultrasound system (FUJIFILM Sonosite, Washington, DC, USA) equipped with a high-resolution linear probe and Sonocal software for automatic measurements. A 10 mm segment of the common carotid artery, 1 cm proximal to the bifurcation, was analysed. Measurements were obtained in lateral, anterior and posterior projections on both near and far walls. Participants were examined in the dorsal decubitus position with cervical extension and turned towards the contralateral shoulder [28].

##### Vascular Function

Vascular function was assessed by cfPWV, baPWV and CAVI.

cfPWV assessments were performed using a SphygmoCor device (AtCor Medical, West Ryde, Australia) while subjects rested in a supine position. The transit time was calculated based on the latency between the electrocardiogram R-wave and the respective carotid and femoral pulse waves. A standard tape measure was utilised to determine the surface distance from the suprasternal notch to both arterial recording points [29]. (Fukuda Denshi, Tokyo, Japan). We placed electrodes on the resting participant’s arms and ankles, and positioned a phonocardiographic microphone at the second intercostal space. We calculated CAVI from the stiffness parameter β as follows:

$$\beta =2\rho \times \frac{1}{2}\left(\alpha +\beta \right)\cos\frac{1}{2}\left(\alpha -\beta \right)$$

where ρ is blood density and PWV is measured between the aortic valve and the ankle. Measurements were considered valid after three consecutive normal heartbeats [30]. baPWV was estimated as: baPWV = (0.5934 × height (cm) + 14.4724)/tba, where tba is the time interval between brachial and ankle waveforms [31].

##### Definition of Accelerated Vascular Ageing

Accelerated vascular ageing (AVA) and normal vascular ageing (NVA) were defined using age- and sex-specific percentiles of the vascular ageing index (VAI) within the study population [32]. The vascular ageing index (VAI) was computed using cfPWV and cIMT with the formula: VAI = (log(1.09) × 10 × cIMT + log(1.14) × cfPWV) × 39.1 + 4.76. Values ≥ 75th percentile were classified as AVA and values < 75th percentile as NVA.

#### 2.3.3. Diagnostic Criteria for Metabolic Syndrome

The diagnosis of MetS was established following the guidelines of the National Cholesterol Education Program Adult Treatment Panel III consensus [11]. Individuals were categorised as having the syndrome if they exhibited a minimum of three out of the five criteria detailed below (Table 1).

#### 2.3.4. Analysis Groups

To investigate how various combinations of MetS components affect arterial stiffness, participants were categorised into four groups: (0) no MetS components; (1) MetS-hypertension: blood pressure component present; (2) MetS-dyslipidaemia: low HDL-cholesterol and/or elevated triglycerides; (3) MetS-increased insulin resistance: elevated fasting glucose and abdominal obesity.

#### 2.3.5. Cardiovascular Risk Factors

Three systolic blood pressure (SBP) and diastolic blood pressure (DBP) measurements were taken using an OMRON M10-IT sphygmomanometer (Omron Healthcare, Kyoto, Japan), and the mean of the last two measurements was recorded. Measurements followed European Society of Hypertension recommendations [12]. Hypertension was defined by a blood pressure reading of ≥140/90 mmHg or the active use of antihypertensive medication.

#### 2.3.6. Laboratory Tests

Blood collection took place in the morning (08:00–09:00 h) after subjects had fasted overnight. Furthermore, a strict 12 h abstinence from smoking, caffeine, and alcoholic beverages was mandated prior to extraction. We quantified serum levels of fasting glucose, total cholesterol, LDL and HDL fractions, and triglycerides. The clinical classifications were defined as follows:

- **Diabetes:** Current use of glucose-lowering pharmacological agents or fasting plasma glucose ≥ 126 mg/dL.
- **Dyslipidaemia:** Prescription of lipid-lowering therapy, total cholesterol ≥ 200 mg/dL, triglycerides ≥ 150 mg/dL, or reduced HDL-cholesterol (<40 mg/dL in men; <50 mg/dL in women).

#### 2.3.7. Anthropometric Measurements

Barefoot body weight (kg) and height (cm) were recorded using a calibrated scale and a Seca 222 stadiometer (Birmingham, UK). Body mass index (BMI) was computed as weight divided by height squared (kg/m^2^), with general obesity established at a BMI ≥ 30 kg/m^2^. In accordance with guideline recommendations, waist circumference (WC) was measured in a standing position, directly above the iliac crests at the end of a forced expiration. Abdominal obesity was categorised as a WC ≥ 102 cm in males and ≥88 cm in females [33].

### 2.4. Statistical Analysis

Quantitative data are expressed as the mean ± standard deviation, while qualitative variables are detailed using absolute counts and percentages. All numerical data were initially tested for normal distribution. When comparing two independent groups, we applied Student’s *t*-test for parametric variables and the Mann–Whitney U test for non-parametric ones. For comparisons across three or more categories, an analysis of variance (ANOVA) or the Kruskal–Wallis test was utilised, contingent upon data normality. Proportional differences in categorical variables were evaluated via the chi-square (χ^2^) test. Finally, to contrast the subgroup lacking any MetS components against the other groups, a Kruskal–Wallis test was conducted, followed by Dunn’s post hoc analysis with a Bonferroni correction.

Associations between vascular measures and MetS (number of components and each component) were assessed using multiple linear regression models with cIMT, cfPWV, baPWV, CAVI and VAI as dependent variables and age in years; sex (men = 0, females = 1); lifestyle: alcohol consumption in gr/week; MD score; smoking status (non-smoker = 0; smoker = 1); number of steps per week; and consumption or not of hypotensive, lipid-lowering, hypoglycaemic and antithrombotic drugs (non-consumption = 0; consumption = 1). Logistic regression models evaluated associations between MetS (and each component) and high vascular measures (≥75th percentile vs. <75th percentile) for cIMT, cfPWV, baPWV and CAVI, adjusted for the aforementioned factors. Data analysis was carried out via SPSS software v28.0 (IBM Corp., Armonk, NY, USA) and R (version 4.4.2), where an α-level of 0.05 was adopted to determine statistical significance.

### 2.5. Ethics

The project was approved by the Committee of Ethics of Research with Medicines of the Health Area of Salamanca on 27 June 2022 (CEIm reference: PI 2022 06 1048). All participants provided written informed consent prior to study participation. Prior to enrolment, all individuals provided written informed consent. This observational study complied with the Declaration of Helsinki [34] and WHO standards. Furthermore, participant data were coded and managed in full compliance with both Spanish legislation and the EU General Data Protection Regulation (GDPR).

## 3. Results

### 3.1. Baseline Clinical Profile of the Participants

Table 2 presents general characteristics, conventional cardiovascular risk factors, lifestyle and medication use, MetS components and parameters of vascular structure, function, and ageing, both overall and stratified by sex. The mean age of the participants was 52.8 ± 11.9 years, and 68% of the cohort was female. Male participants were older and exhibited a higher prevalence of arterial hypertension, diabetes mellitus, dyslipidaemia, and general obesity. Males showed a higher prevalence of medication use and consumption of alcohol compared to females. The proportion with MetS and the components of elevated blood pressure, elevated fasting glucose and elevated triglycerides was higher in males. All vascular parameters were higher in males than females.

From the date of infection by the virus and the date of inclusion of each subject in the study, 38.7 ± 9.6 months elapsed. No statistically significant differences were observed between sexes.

**Figure 2:**
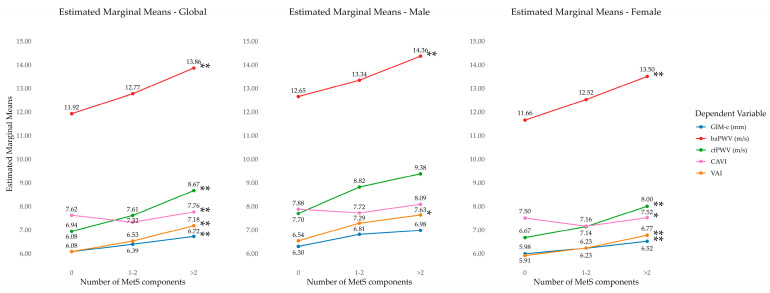
Age-adjusted marginal means for parameters of vascular structure, vascular function and vascular ageing measured by the number of MetS components (overall and by sex). MetS, metabolic syndrome; cIMT, carotid intima–media thickness; cfPWV, carotid–femoral pulse wave velocity; baPWV, brachial–ankle pulse wave velocity; CAVI, cardio–ankle vascular index; VAI, vascular ageing index. * *p* < 0.05; ** *p* < 0.01.

Figure 2 illustrates age-adjusted marginal means for parameters of vascular structure, function and vascular ageing by increasing the number of MetS components overall and by sex. Overall, and in females, all measures increased with a higher number of MetS components; in men, trends were similar but statistically significant mainly for cIMT and the VAI.

**Figure 3:**
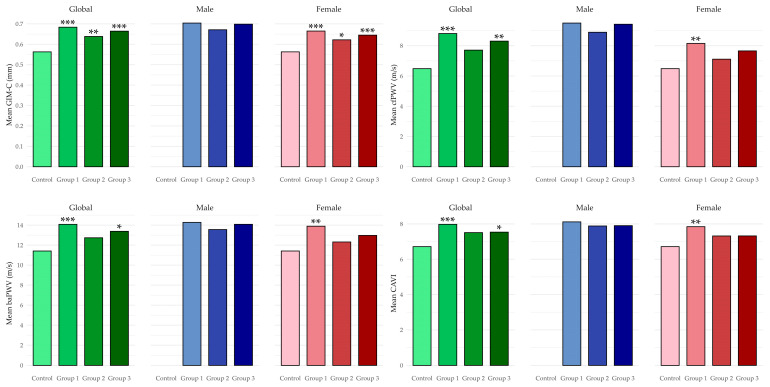
Measured values of vascular structure and function parameters by group (overall and by sex). Control group: participants without any MetS components. Group 1 (MetS-hypertension): blood pressure component. Group 2 (MetS-dyslipidaemia): low HDL-cholesterol and/or elevated triglycerides. Group 3 (MetS-increased insulin resistance): elevated fasting glucose and abdominal obesity. Abbreviations as above. Differences versus control: * *p* < 0.05; ** *p* < 0.01; *** *p* < 0.001.

Figure 3 shows measured values of vascular structure and function by MetS component groups overall and by sex. Differences are compared with the group with no MetS components. In men, between-group comparisons were not performed because no males were present in the group without any MetS components.

#### Degree of Vascular Ageing According to the VAI

Table 3 shows the values of cardiovascular risk factors, MetS components, and vascular structure and function parameters according to whether they are classified as AVA or NVA with VAI percentiles. Subjects with hypertension, diabetes, and obesity are classified in a higher percentage to AVA and have a higher number of components and percentage of subjects with MetS, and higher values of vascular structure and function parameters. The same data are shown for males and females in Tables S1 and S2 of the Supplementary Materials.

Values are means ± standard deviation for continuous data and number and proportions for categorical data. VAI: vascular ageing index; BMI: body mass index; WC: waist circumference; TGC: triglycerides. cIMT: intima–media thickness of common carotid; cfPWV: carotid–femoral pulse wave velocity; baPWV: brachial–ankle pulse wave velocity; CAVI: cardio–ankle vascular index. *p* value: differences between subjects with and without vascular ageing.

### 3.2. Multiple Linear Regression: Associations Between Vascular Measures and MetS Components

Table 4 shows associations between vascular measures and the number of MetS components and each component. All vascular measures show a positive association with artery pressure. With the exception of CAVI, all measured parameters showed a positive association with the number of MetS components. Furthermore, cIMT, cfPWV, and the VAI were positively associated with waist circumference. baPWV was positively associated with all MetS components except HDL-cholesterol, which showed an inverse association. CAVI was inversely associated with waist circumference.

**Figure 4:**
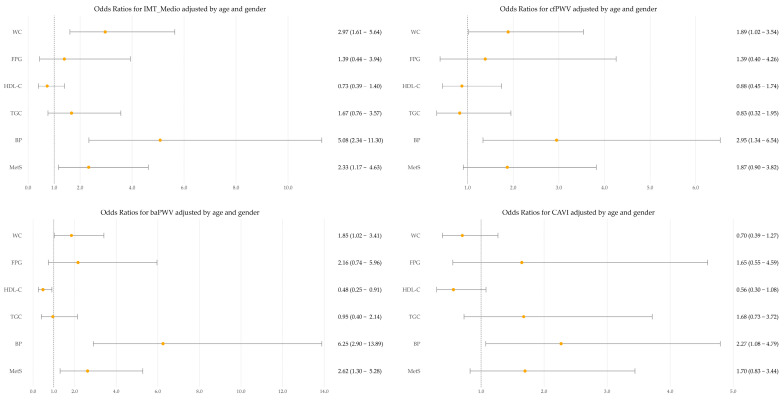
Logistic regression analysis of high vascular measures (≥75th percentile) for cIMT, cfPWV, baPWV and CAVI in relation to MetS and its components, adjusted for age, sex, lifestyle and drug use. MetS, metabolic syndrome; BP, blood pressure; TGC, triglycerides; HDL, high-density lipoprotein; FPG, fasting plasma glucose; WC, waist circumference.

Figure 4 presents logistic regression analyses for high vascular measures (≥75th percentile). cIMT showed odds ratios > 1 with the number of MetS components and with the blood pressure and waist circumference components. cfPWV showed odds ratios > 1 with blood pressure and waist circumference. baPWV showed odds ratios > 1 with the number of MetS components, with blood pressure and waist circumference, and an odds ratio < 1 with HDL-cholesterol. CAVI showed no significant association with MetS components.

## 4. Discussion

In this sample of adults with long COVID, the presence of metabolic syndrome (23.7%) and, in particular, the accumulation of its components were consistently associated with a worse vascular profile. Specifically, arterial stiffness (cfPWV and baPWV), carotid intima–media thickness and the vascular ageing index (VAI) increased as the number of MetS components rose. Overall, these findings suggest that MetS may act as an amplifier of subclinical vascular damage in the context of long COVID.

From a pathophysiological perspective, long COVID has been linked to persistent endothelial activation, low-grade inflammation and a residual prothrombotic state even months after acute infection, which may promote arterial stiffness and vascular remodelling [4,6]. MetS shares convergent mechanisms—insulin resistance, chronic inflammation, oxidative stress and endothelial dysfunction—that accelerate vascular ageing [8,10,13,14,15]. Therefore, a co-occurrence of long COVID and MetS could exert a synergistic effect on vascular deterioration, consistent with the graded associations observed between the number of MetS components and measures of vascular structure, function and ageing.

Component-level analyses provided clinically relevant information. Blood pressure and central adiposity showed particularly robust associations with cfPWV, baPWV and the VAI, reinforcing their role as key determinants of vascular impairment in both general and cardiometabolic populations. These results are consistent with prior studies identifying hypertension and abdominal obesity as major drivers of arterial ageing [12,14,17].

In this study, we found a positive association of triglycerides and a negative association of HDL-cholesterol with baPWV alone. This result is not consistent with the evidence linking hypertriglyceridaemia with an atherogenic lipoprotein profile, vascular inflammation, and increased arterial stiffness with other parameters [12,14,17]. The negative association of HDL-cholesterol showed an inverse association with baPWV, consistent with its cardioprotective role and previous observations that higher HDL-cholesterol is associated with better arterial elasticity and less peripheral stiffness [13,15].

An important finding was the behaviour of CAVI, which showed lower sensitivity to the metabolic burden gradient and inverse associations with waist circumference. This pattern has been reported previously and may relate to CAVI’s lower direct dependence on blood pressure and to the influence of body composition on its estimation [17,32]. These methodological differences between indices suggest that, in long COVID, combining central stiffness (cfPWV), peripheral stiffness (baPWV) and structural markers (cIMT) provides a more comprehensive characterisation of the vascular tree than relying on a single measure.

Sex differences—a higher MetS prevalence and worse vascular parameters in men—are consistent with patterns observed in the general population and may be mediated by differences in hormones, fat distribution and accumulated cardiovascular risk burden [10,12]. However, the larger proportion of females and the cross-sectional design warrant caution when interpreting sex-stratified analyses. Longitudinal studies with larger samples are needed to clarify sex-specific vascular ageing trajectories in long COVID.

Clinically, our results support systematic cardiometabolic assessments in patients with long COVID, with particular attention to abdominal obesity and blood pressure. Lifestyle interventions—weight reduction, regular physical activity and a cardioprotective diet—together with optimal control of blood pressure, glucose and lipids, are recommended strategies to reduce cardiometabolic risk and may help attenuate vascular deterioration in this population [12,13,16], although this hypothesis should be confirmed in longitudinal and interventional studies. We should not forget that, although our findings highlight a clear link between MetS and arterial stiffness in long COVID patients, the lack of a comparative cohort of individuals without long COVID precludes the determination of whether this vascular impairment is specifically exacerbated by the post-viral state or reflects the baseline cardiovascular risk of individuals with MetS.

Our study has several limitations. First, its cross-sectional design prevents us establishing causal relationships. Second, the absence of a control group of subjects without long COVID (either healthy individuals or those who recovered fully from COVID-19) limits our ability to estimate the specific attributable risk of long COVID on vascular health. Consequently, our results should be interpreted as a characterisation of the vascular status in a long COVID population with MetS, rather than a direct comparison of the effect of the infection itself. Furthermore, while chronic inflammation is a hypothesised driver of vascular damage in both MetS and long COVID, our study did not include systemic inflammatory biomarkers such as high-sensitivity C-reactive protein (Hs-CRP) or Red Cell Distribution Width (RDW). Future studies should incorporate these markers to further elucidate the inflammatory pathways through which long COVID may interact with metabolic dysregulation to promote vascular ageing. Nonetheless, the consistency of results across analyses and the use of multiple vascular indicators strengthen the findings.

## 5. Conclusions

In conclusion, among adults with long COVID, metabolic syndrome and the accumulation of its components are associated with poorer vascular structure, function, and accelerated vascular ageing. Although the absence of a control group limits the estimation of risk specifically attributable to long COVID, these findings underscore the importance of metabolic health management in the clinical follow-up of these patients and support the need for longitudinal studies to establish temporality, mechanisms and intervention impact in this vulnerable population.

## Figures and Tables

**Table 1:** Diagnostic criteria for MetS.

Component	Criterion
**Abdominal obesity (WC)**	≥102 cm (males) or ≥88 cm (females)
**Serum triglycerides**	≥150 mg/dL, or receiving specific pharmacological treatment
**High-density lipoprotein cholesterol**	Less than 40 mg/dL in males and less than 50 mg/dL in females
**Fasting glycaemia**	≥100 mg/dL or receiving glucose-lowering drugs
**Arterial pressure**	Systolic ≥ 130 mmHg and/or diastolic ≥ 85 mmHg, or use of blood pressure medication

Adapted from NCEP ATP III [11].

**Table 2:** Baseline clinical profile of the participants.

Variable	Overall (*n* = 304)	Males (*n* = 97)	Females (*n* = 207)	*p* Value
Lifestyle				
Smoking status, *n* (%)	16 (5.3)	8 (8.2)	8 (3.9)	0.111
Alcohol consumption, (gr/week)	29.32 ± 52.93	60.39 ± 76.35	14.76 ± 27.15	<0.001
Physical activity (steps/day)	7448.45 ± 3694.33	7069.35 ± 3373.54	7623.86 ± 3829.03	0.160
Mediterranean Diet score	7.80 ± 2.34	7.71 ± 2.23	7.84 ± 2.39	0.424
Cardiovascular risk factors				
Age (years)	52.78 ± 11.91	55.70 ± 12.28	51.41 ± 11.51	0.003
Systolic blood pressure (mmHg)	119.98 ± 16.77	129.45 ± 14.37	115.53 ± 15.98	<0.001
Diastolic blood pressure (mmHg)	76.88 ± 11.12	82.34 ± 11.04	74.33 ± 10.22	<0.001
Arterial hypertension, *n* (%)	109 (35.9)	52 (53.6)	57 (27.5)	<0.001
Total cholesterol (mg/dL)	187.43 ± 34.35	182.11 ± 32.94	189.93 ± 34.80	0.065
Low-density lipoprotein cholesterol (mg/dL)	113.09 ± 31.80	113.59 ± 32.12	112.86 ± 31.72	0.852
High-density lipoprotein cholesterol (mg/dL)	56.84 ± 13.53	48.78 ± 10.86	60.64 ± 13.02	<0.001
Triglycerides (mg/dL)	102.27 ± 50.89	117.47 ± 54.39	95.12 ± 47.64	<0.001
LN Triglycerides (mg/dL)	4.53 ± 0.44	4.68 ± 0.42	4.46 ± 0.43	<0.001
Fasting plasma glucose (mg/dL)	87.91 ± 17.69	94.37 ± 19.78	84.87 ± 15.76	<0.001
BMI (kg/m^2^)	27.99 ± 5.55	29.60 ± 4.64	27.23 ± 5.79	<0.001
Waist circumference (cm)	93.92 ± 15.49	104.34 ± 12.52	89.04 ± 14.31	<0.001
MetS, *n* (%)	72 (23.7)	39 (40.2)	33 (15.9)	<0.001
Medication *n* (%)				
Antihypertensive therapy, *n* (%)	79 (26.0%)	34 (35.1%)	45 (21.7%)	0.014
Lipid-lowering drugs, *n* (%)	75 (24.7%)	40 (41.2%)	35 (16.9%)	<0.001
Antithrombotic drugs, *n* (%)	17 (5.6%)	9 (9.3%)	8 (3.9%)	0.056
Glucose-lowering drugs, *n* (%)	32 (10.5%)	18 (18.6%)	14 (6.8%)	0.002
Vascular structure and function				
cIMT (mm)	0.64 ± 0.09	0.68 ± 0.12	0.62 ± 0.07	<0.001
cfPWV (m/s)	7.67 ± 2.36	8.85 ± 2.95	7.12 ± 1.79	<0.001
baPWV (m/s)	12.79 ± 2.38	13.63 ± 2.40	12.40 ± 2.27	<0.001
CAVI	7.51 ± 1.26	7.90 ± 1.36	7.33 ± 1.17	<0.001
VAI	65.54 ± 13.75	72.89 ± 16.66	62.13 ± 10.59	<0.001

Abbreviations: BMI, body mass index; MetS, metabolic syndrome; cIMT, carotid intima–media thickness; cfPWV, carotid–femoral pulse wave velocity; baPWV, brachial–ankle pulse wave velocity; CAVI, cardio–ankle vascular index; VAI, vascular ageing index. *p* value: differences between males and females.

**Table 3:** Cardiovascular risk factors and MetS components according to vascular ageing status (overall).

	With VAI (*n* = 73)		Without VAI (*n* = 230)		*p* Value
	Mean or n°	SD or (%)	Mean or n°	SD or (%)	
Males, *n* (%)	23	31.5%	73	31.7%	0.970
Females, *n* (%)	50	68.5%	157	68.3%	
Evolution time, months	38.07	10.05	38.84	9.45	0.553
Age (years)	54.89	10.37	52.07	12.32	0.079
Nº. of cigarettes (day)	18.42	15.29	14.90	9.04	0.767
Smoker, *n* (%)	4	4.8%	11	5.5%	0.811
Systolic blood pressure (mmHg)	128.77	17.63	117.14	15.53	<0.001
Diastolic blood pressure (mmHg)	82.08	11.91	75.19	10.34	<0.001
Hypertension, *n* (%)	42	57.5%	67	29.1%	<0.001
Antihypertensive therapy, *n* (%)	30	41.1%	49	21.3%	<0.001
Total cholesterol, (mg/dL)	194.92	37.77	185.28	32.80	0.036
Low-density lipoprotein cholesterol (mg/dL)	120.30	34.52	110.99	30.54	0.029
High-density lipoprotein cholesterol (mg/dL)	55.51	14.18	57.31	13.34	0.201
Triglycerides (mg/dL)	110.33	53.92	99.76	49.84	0.063
LN Triglycerides (mg/dL)	4.61	0.41	4.50	0.44	0.063
Dyslipidaemia, *n* (%)	49	67.1%	130	56.5%	0.108
Lipid-lowering drugs, *n* (%)	14	19.2%	60	26.1%	0.231
FPG, (mg/dL)	92.68	24.87	86.39	14.48	0.015
Diabetes mellitus, *n* (%)	15	20.5%	21	9.1%	0.009
Hypoglycaemic drugs, *n* (%)	8	11.0%	39	17.0%	0.217
Weight, kg	82.55	17.51	73.88	16.86	<0.001
Height, cm	165.50	9.33	164.20	8.53	0.448
BMI, (kg/m^2^)	30.11	5.88	27.29	5.28	<0.001
WC, cm	99.51	15.11	92.04	15.14	<0.001
Obesity, *n* (%)	34	46.6%	64	27.8%	0.003
MetS and its components					
Number of MetS components	2.10	1.20	1.35	1.33	<0.001
MetS, *n* (%)	27	37.0%	44	19.1%	0.002
BP ≥ 130/85 mmHg, *n* (%)	52	71.2%	91	39.6%	<0.001
Fasting plasma glucose ≥ 100 mg/dL, *n* (%)	19	26.0%	33	14.4%	0.022
TGC ≥ 150 mg/dL, *n* (%)	13	17.8%	30	13.1%	0.316
HDL-C mg/dL <40 males, <50 mg/dL females, *n* (%)	19	26.0%	51	22.3%	0.508
Elevated WC (≥88 cm females, ≥102 cm males), *n* (%)	50	68.5%	106	46.1%	<0.001
Vascular structure, function, and vascular ageing					
cIMT, mm	0.68	0.08	0.62	0.09	<0.001
cfPWV, m/sec	10.09	2.83	6.90	1.54	<0.001
baPWV, m/sec	14.30	2.79	12.31	2.01	<0.001
CAVI	7.85	1.42	7.39	1.18	0.014

Values are means ± standard deviation for continuous data and number and proportions for categorical data. VAI: vascular ageing index; BMI: body mass index; WC: waist circumference; TGC: triglycerides. cIMT: intima–media thickness of common carotid; cfPWV: carotid–femoral pulse wave velocity; baPWV: brachial–ankle pulse wave velocity; CAVI: cardio–ankle vascular index. *p* value: differences between subjects with and without vascular ageing.

**Table 4:** Association of parameters of vascular structure, vascular function and vascular ageing with the metabolic syndrome and its components with multiple regression analysis.

cIMT, mm	β	(IC	95%)	*p*
N° MetS components	0.014	0.007	0.022	<0.001
Systolic blood pressure (mmHg)	0.002	0.001	0.002	<0.001
Diastolic blood pressure (mmHg)	0.002	0.001	0.003	<0.001
Fasting plasma glucose (mg/dL)	0.001	0.000	0.001	0.089
Triglycerides (mg/dL)	0.010	−0.011	0.031	0.337
High-density lipoprotein cholesterol (mg/dL)	0.000	−0.001	0.000	0.187
Waist circumference, cm	0.001	0.001	0.002	<0.001
cfPWV, m/sec				
N° MetS components	0.218	0.024	0.413	0.027
Systolic blood pressure (mmHg)	0.036	0.022	0.051	<0.001
Diastolic blood pressure (mmHg)	0.044	0.023	0.064	<0.001
Fasting plasma glucose (mg/dL)	0.001	−0.014	0.016	0.857
Triglycerides (mg/dL)	0.278	−0.251	0.807	0.302
High-density lipoprotein cholesterol (mg/dL)	0.003	−0.015	0.021	0.757
Waist circumference, cm	0.022	0.005	0.038	0.009
baPWV, m/seg				
N° MetS components	0.429	0.242	0.615	<0.001
Systolic blood pressure (mmHg)	0.055	0.042	0.069	<0.001
Diastolic blood pressure (mmHg)	0.070	0.051	0.089	<0.001
Fasting plasma glucose (mg/dL)	0.020	0.005	0.034	0.008
Triglycerides (mg/dL)	0.574	0.056	1.092	0.030
High-density lipoprotein cholesterol (mg/dL)	−0.021	−0.039	−0.003	0.025
Waist circumference, cm	0.016	0.000	0.033	0.050
CAVI				
N° MetS components	0.024	−0.079	0.126	0.651
Systolic blood pressure (mmHg)	0.012	0.004	0.020	0.002
Diastolic blood pressure (mmHg)	0.021	0.010	0.031	<0.001
Fasting plasma glucose (mg/dL)	0.006	−0.001	0.014	0.108
Triglycerides (mg/dL)	0.031	−0.248	0.309	0.829
High-density lipoprotein cholesterol (mg/dL)	0.001	−0.009	0.010	0.913
Waist circumference, cm	−0.011	−0.019	−0.002	0.017
VAI				
N° MetS components	1.595	0.553	2.637	0.003
Systolic blood pressure (mmHg)	0.239	0.163	0.314	<0.001
Diastolic blood pressure (mmHg)	0.286	0.176	0.395	<0.001
Fasting plasma glucose (mg/dL)	0.026	−0.055	0.107	0.535
Triglycerides (mg/dL)	1.789	−1.072	4.649	0.219
High-density lipoprotein cholesterol (mg/dL)	−0.001	−0.101	0.098	0.978
Waist circumference, cm	0.157	0.068	0.246	<0.001

Multiple regression analysis using as dependent variable vascular structure parameters (GIMc), vascular function (cfPWV; baPWV and CAVI) and vascular ageing (VAI). As independent variables: number of MetS components, systolic blood pressure, diastolic blood pressure, fasting plasma glucose, triglycerides, high-density lipoprotein cholesterol and waist circumference. As adjustment variables: age, sex, lifestyle and drug use. MetS, metabolic syndrome; cIMT: intima–media thickness of common carotid; cfPWV: carotid–femoral pulse wave velocity; baPWV: brachial–ankle pulse wave velocity; CAVI: cardio–ankle vascular index; VAI: vascular ageing index.

## Data Availability

The data supporting the findings of this study are available on ZENODO at: https://doi.org/10.5281/zenodo.14282873.

## Supplementary Materials

The following supporting information can be downloaded at: https://www.mdpi.com/article/10.3390/jcm15062348/s1, Table S1: Cardiovascular risk factors and MetS components according to vascular ageing in males; Table S2: Cardiovascular risk factors and MetS components according to vascular ageing in females.

## Abbreviations

The following abbreviations are used in this manuscript:

MetS	metabolic syndrome
PWV	Pulse wave velocity
CfPWV	carotid–femoral pulse wave velocity
cIMT	carotid intima–media thickness
BaPWV	brachial–ankle pulse wave velocity
CAVI	cardio–ankle vascular index
VAI	vascular ageing index
APISAL	Primary Care Research Unit of Salamanca
AVA	Accelerated vascular ageing
NVA	normal vascular ageing
WC	waist circumference
HDL	high-density lipoprotein
SBP	systolic blood pressure
DBP	diastolic blood pressure
HDL-C	High-density lipoprotein
BMI	Body mass index
