# Supplementary material for: Association Between Metabolic Syndrome Components and Vascular Structure and Function in Subjects with a Diagnosis of Long COVID: The BioICOPER Study
Source: J Clin Med. 2026 Mar 19;15(6):2348. doi: 10.3390/jcm15062348 (PMC13027218; doi:10.3390/jcm15062348)
Supplement: Supplementary file 1 [file jcm-15-02348-s001.zip › jcm-4189259-supplementary.pdf]

Table S1. Differences in cardiovascular risk factors and components of MetS between subjects with and without Vascular Ageing in males.

	**With VAI (*n*= 23)**		**Without, VAI (*n*=73)**		***p* Value**
Conventional risk factors	Mean or n°	SD or (%)	Mean or n°	SD or (%)	
Age, (years)	56.61	12.52	55.36	12.35	0.673
N° Cigarettes (day)	21.70	13.82	19.30	9.75	0.868
Smoker, n (%)	1	4.3%	6	8.2%	0.533
SBP, (mmHg)	137.22	14.19	127.00	13.73	0.006
DBP, (mmHg)	87.30	12.26	80.71	10.29	0.012
Hypertension, n (%)	16	69.6%	36	49.3%	0.089
Antihypertensive drugs, n (%)	10	43.5%	24	32.96%	0.354
Total cholesterol, (mg/dl)	194.57	35.06	178.89	31.22	0.044
LDL cholesterol, (mg/dl)	124.70	31.48	110.73	31.54	0.067
HDL cholesterol, (mg/dl)	48.83	10.81	48.79	11.02	0.908
Triglycerides, (mg/dl)	128.39	55.59	114.42	54.24	0.189
Dyslipidemia, n (%)	18	78.3%	51	69.9%	0.435
Lipid–lowering drugs. n (%)	5	21.7%	34	46.6%	0.034
FPG, (mg/dl)	95.09	22.91	94.23	18.98	0.747
Diabetes mellitus, n (%)	5	21.7%	16	21.9%	0.986
Hypoglycaemic drugs, n (%)	1	4.3%	5	6.8%	0.666
Weight, kg	91.43	14.14	87.00	15.24	0.154
Height, cm	175.72	6.08	171.56	7.50	0.021
BMI, (kg/m^2)	29.58	4.11	29.57	4.85	0.847
WC, cm	104.59	11.74	104.06	12.80	0.585
Obesity, n (%)	11	47.8%	32	43.8%	0.737
MetS and its components					
Number of components MetS	2.26	1.10	1.96	1.46	0.324
MetS, n (%)	10	43.5%	28	38.4%	0.661
BP ≥ 130/85 mmHg, n (%)	20	87.0%	49	67.1%	0.065

FPG ≥ 100 mg/dL, n (%)	8	34.8%	23	31.5%	0.770
TGC ≥150 mg/dL, n (%)	6	26.1%	15	20.5%	0.575
HDL-C mg/dl <40 males, <50 mg/dl females, n (%)	4	17.4%	19	26.0%	0.397
WC ≥88 cm females, ≥102 cm males, n (%)	14	60.9%	37	50.7%	0.393
Vascular Structure, Function, and Vascular Ageing					
GIM-c, mm	0.71	0.10	0.67	0.12	0.069
cfPWV, m/sec	12.15	3.69	7.79	1.67	<0.001
baPWV, m/seg	15.02	2.82	13.15	2.07	0.006
CAVI	8.26	1.54	7.76	1.28	0.121

Values are means standard deviations for continuous data and number and proportions for categorical data. VAI: Vascular Aging Index; SBP: systolic blood pressure; DBP: diastolic blood pressure; LDL: low–density lipoprotein; HDL: high–density lipoprotein; FPG: fasting plasma glucose; BMI: body mass index; WC Waist circumference; TGC: Triglycerides. c-IMT: Intima–media thickness of common carotid; cf-PWV: carotid-femoral pulse wave velocity; baPWV: Brachial-ankle pulse wave velocity; CAVI: Cardio-ankle vascular index. *p* value: differences between subjects with and without vascular Ageing.

Table S2. Differences in cardiovascular risk factors and components of MetS between subjects with and without Ageing vascular in Females .

	**With VAI (*n*=50)**		**Without, VAI (*n*=157)**		***p* Value**
Conventional risk factors	Mean or nº	SD or (%)	Mean or n°	SD or (%)	
Age, (years)	54.10	9.25	50.55	12.04	0.057
N° Cigarrillos (day)	16.38	16.24	12.09	7.35	0.868
Smoker, n (%)	3	6.0%	5	3.2%	0.368
SBP, (mmHg)	124.88	17.81	112.56	14.16	<0.001
DBP, (mmHg)	79.68	11.07	72.62	9.34	<0.001
Hypertension, n (%)	26	52.0%	31	19.7%	<0.001
Antihypertensive drugs, n (%)	20	40.0%	25	15.9%	<0.001
Total cholesterol, (mg/dl)	195.08	39.30	188.28	33.20	0.230
LDL cholesterol, (mg/dl)	118.27	35.95	111.12	30.16	0.166
HDL cholesterol, (mg/dl)	58.58	14.57	61.29	12.46	0.138
Triglycerides, (mg/dl)	102.02	51.59	92.90	46.25	0.131

Dyslipidemia, n (%)	31	62.0%	79	50.3%	0.149
Lipid–lowering drugs. n (%)	9	18.0%	26	16.6%	0.813
FPG, (mg/dl)	91.58	25.87	82.72	9.92	0.011
Diabetes mellitus, n (%)	10	20.0%	5	3.2%	<0.001
Hypoglycaemic drugs, n (%)	7	14.0%	34	21.7%	0.237
Weight, kg	78.46	17.51	67.78	13.85	<0.001
Height, cm	160.80	6.30	160.78	6.62	0.745
BMI, (kg/m^2)	30.36	6.57	26.23	5.15	<0.001
WC, cm	97.17	16.00	86.45	12.72	<0.001
Obesity, n (%)	23	46.0%	32	20.4%	<0.001
MetS and its components					
Number of components MetS	2.02	1.25	1.07	1.17	<0.001
MetS, n (%)	17	34.0%	16	10.2%	<0.001
BP ≥ 130/85 mmHg, n (%)	32	64.0%	42	26.8%	<0.001
FPG ≥ 100 mg/dL, n (%)	11	22.0%	10	6.4%	0.002
TGC ≥150 mg/dL, n (%)	7	14.0%	15	9.6%	0.382
HDL-C mg/dl <40 males, <50 mg/dl females, n (%)	15	30.0%	32	20.5%	0.164
WC ≥88 cm females, ≥102 cm males, n (%)	36	72.0%	69	43.9%	<0.001
Estructura, función vascular y envejecimiento vascular					
GIM-c, mm	0.67	0.08	0.60	0.06	<0.001
cfPWV, m/sec	9.14	1.66	6.48	1.29	<0.001
baPWV, m/seg	13.96	2.74	11.91	1.86	<0.001
CAVI	7.66	1.33	7.22	1.10	0.020

Values are means standard deviations for continuous data and number and proportions for categorical data. VAI: Vascular Aging Index;; SBP: systolic blood pressure; DBP: diastolic blood pressure; LDL: low–density lipoprotein; HDL: high–density lipoprotein; FPG: fasting plasma glucose; BMI: body mass index; WC Waist circumference; TGC: Triglycerides. c-IMT: Intima–media thickness of common carotid; cf-PWV: carotid-femoral pulse wave velocity; baPWV: Brachial-ankle pulse wave velocity; CAVI: Cardio-ankle vascular index. *p* value: differences between subjects with and without vascular Ageing.
